# Editorial: Psychological experiences and responses in the global south amidst and ahead of the COVID-19 pandemic

**DOI:** 10.3389/fpsyg.2023.1172853

**Published:** 2023-06-13

**Authors:** Nelesh Dhanpat, Madelyn Geldenhuys, Shaun Ruggunan

**Affiliations:** ^1^Department of Industrial Psychology and People Management, College of Business and Economics, University of Johannesburg, Johannesburg, South Africa; ^2^School of Arts and Sciences, Discipline of Psychology, The University of Notre Dame, Sydney, NSW, Australia; ^3^School of Management, Information Technology and Governance, College of Law, Management and Commerce, University of KwaZulu Natal, Durban, South Africa

**Keywords:** COVID-19, workplace, remote work, employee wellbeing, employee experience

## Introduction

The COVID-19 pandemic caused unprecedented disruption across all levels of society and businesses, with significant implications for individual lives and work (Ainamani et al., 2020). The debilitating effects of the pandemic posed significant risks to individual health and economic wellbeing (Danquah et al., 2020) and resulted in many changes in how we work. With worldwide governments imposing various quarantine measures (e.g., lockdowns, isolation, and mask-wearing) to control the spread of the virus (Chowdhury and Jomo, 2020; Guan et al., 2020), organizations also had to implement work from home (WFH) measures to help mitigate the spread of the virus, causing employees to adjust with limited resources (De Bruin et al., 2020; Dhanpat et al., 2022).

As the pandemic significantly impacted individuals' lives and work, it has led to several psychological challenges. For example, a high degree of uncertainty, anxiety, and stress, especially in the context of work, developed resulting in several psychological issues (Lu et al., 2020) and workplace issues. Additionally, research into the effects of the pandemic and work have predominantly been carried out in the Global North. Although countries in the Northern hemisphere are struggling to manage the effects of the pandemic, most countries in the Southern hemisphere are extremely resource poor, have high levels of inequality but are also more experienced in dealing with previous pandemics such as HIV. Nonetheless, under resourced countries may find adjusting to life and work after the pandemic to be more challenging (see Sow, 2022). The work presented in this Research Topic is located in the southern hemisphere and mostly in the global south. Conceptually, we recognize that these are two different terms, the former referring to countries located in the southern hemisphere and the latter referring to the least industrialized countries, the majority of which are located in the southern hemisphere. In both instances, such work is underrepresented in the literature on the psychological experiences of the COVID-19 pandemic. As such, understanding the *psychological experiences and responses in the Global South amidst and ahead of the COVID-19 pandemic* can enable organizations to adjust amidst the problems they are facing.

As guest editors to this special edition, we accepted 15 articles that investigated several of the psychological experiences and responses amidst and ahead for the COVID-19 pandemic in the global south (combined sample size of 3497). Organized into themes, we believe that the findings of each of these research endeavors make critical contributions to the literature and to workplaces by understanding the impact COVID-19 had and continues to have on employees and their organizations. It also enables us to understand where to develop research interests for future research. The articles also help fill an empirical gap by drawing attention to national contexts that do not feature significantly in the western dominated literature on the pandemic.

The first theme that emerged is *organizational support and employee engagement* (with a combined sample of 2652). In their research, Reynell van der Ross et al. explored academic staff engagement and burnout risk in a Higher Education setting during the COVID-19 pandemic. Higher education has long faced difficulties in relation academic stress and burnout. Reynell van der Ross et al. found that leaders who prioritize employee enabling creating working conditions may also facilitate staff engagement. Similarly, Dekel et al. explores the relationship between psychological wellbeing, volunteer work engagement, and perceived organizational support among young adult volunteers in not-for-profit organizations in Australia amidst the COVID-19 pandemic and lockdown period. Their research shows that perceived organizational support is critical for the process of psychological wellbeing and volunteer engagement (Dekel et al.). It seems that irrespective of what can be viewed as a traumatic global event, with supporting work environments, employees can still engage in their work. This study assessed the combined impact of technostress, work-family conflict, and perceived organizational support on workplace flourishing for higher education employees during the pandemic, highlighting the need for additional support and policies that prioritize work-life balance (Harunavamwe and Ward). Ronnie et al. found that the psychological contract between women academics and their institutions in South Africa during the pandemic is critical and there seems to be major shifts in workload, resources, communication, trust, and support because of the pandemic. Positive psychological factors during the pandemic had important implications for the wellbeing of people. For example, Sekaja et al. found that manifesting gratitude at work can have many positive outcome for the employee and workplace. For example, reflecting on what employees can be thankful for can be a way of enhancing gratitude and thereby, wellness, performance, and commitment. Ngobeni et al. believes that the psychological contract at work can be crucial in determining the engagement of employees. Their research probes how leaders feel they can influence of the psychological contract on employee engagement. They found that continuous change in the world, exacerbated by the COVID-19 pandemic, can influence employee expectations. This can inadvertently have in impact on the relationship between people's perceptions of the psychological contract on employee engagement (Ngobeni et al.).

With mental health and wellbeing concerns on the rise, a second critical theme emerged, namely *work and mental health* (with a combined sample of 375). In their research, Moralo and Graupner explored the role of industrial psychology practitioners in managing the psychological impact of COVID-19 on employees. They found that Industrial/Organizational psychology practitioner's role in the changing world of work enables organizations to be prepared for the changes by providing multi-level interventions (Moralo and Graupner) and these should be leveraged more. Maharaj and Ramsaroop explored the relationship between emotional intelligence (EQ) and educators' quality of life during the pandemic. They propose that it is EQ as an essential resilience skill for enhancing the quality of life especially during times of adversity. A practical research model was advocated for key stakeholders in the South African basic education sector (Maharaj and Ramsaroop). Comparing the psychological health of previously infected and non-infected South African employees regarding burnout, anxiety, depression, and stress, Hill found that infected participants had significantly higher levels of burnout, anxiety, depression, and stress than non-infected participants. The study recommends industrial psychologists to manage the psychological impact of COVID-19 at work (Hill).

*Remote work and working from home* have become a new way work and it is expected to continue. This third theme (with combined sample of 131) showed that work from home during the pandemic had increased workloads and domestic responsibilities, resulting in significant negative impacts on mental and physical wellbeing (Singh et al.), while Chinyamurindi found remote working had resulting in decreased employee engagement of women leaders in the public service industry. Consequently, this also had long-term negative effects on the industry (Chinyamurindi). It is also expected that hybrid work models will continue to be possible post-pandemic (Smite et al., 2023) and holds advantages for employees such as lower psychological stress responses and associated productivity (Shimura et al., 2021). Although some research shows that work from home can be rewarding and increase performance, more research into work and other life roles seems to be an important area that needs investigating.

*Occupational health education* seems to be an important factor for countries in the global South (combined sample of 38). A study by Mapuranga et al. on mandatory vaccination in Zimbabwe's retail sector found most organizations require vaccination or risk job loss. Four consultation levels exist, with the fourth being most common. Employees had three categories of perceptions and varied reactions, including willingly getting vaccinated, settling for one dose, or fraudulently obtaining cards. Genuine consultation between management and employees is crucial. Meyer et al. describes a teaching approach in occupational health psychology that engages postgraduate students in South Africa as learners, research participants, and co-researchers, using their reflections on their experiences during the first 19 months of the COVID-19 pandemic. The study highlights the importance of considering contextual factors when engaging in occupational health psychology education, research, and practice, and illustrates the use of Sense-Maker as a research tool to develop and implement relevant and effective means to support employee health and wellbeing.

Lastly, *leadership and workplace management* can enable efficient workplaces and performance (combined sample of 301). Meadows and De Braine explores how the COVID-19 pandemic impacted the work identities of leaders and how they (effectively) responded to the challenges. They found that leaders employed virtual leadership to ensure that customers' expectations were met, and to manage team-and organizational performance. They did this by building effective teams and fostering a digital culture, taking on extra roles such as strategist, technology expert, and coach (Meadows and De Braine). The research by Deas and Coetzee observed generational cohort differences regarding value-orientated psychological contract expectations for job characteristics and work-life balance and that this difference could be utilized to develop interventions and strategies to promote retention of employees in the post-pandemic digital-orientated workplace.

## Conclusion

The collection of articles in this Research Topic provides an in-depth analysis of the psychological challenges faced by employees predominantly in the South African context. South Africa remains one the most unequal societies globally, and empirical work on managing people during times of profound crisis in such a context is especially important since South Africa in many respects is representative of the majority world. Extant work on psychological experiences of COVID-19 in the workplace remains dominated by empirical work in the minority world. This Research Topic is a step toward showcasing the diversity of experiences and ways of theorizing in under studied contexts. Future research can examine more comparatively the experiences of the global North and global South in pandemic and post-pandemic workplaces. Research questions that delve into the importance of macro-contextual factors such as political economy, income inequality levels, employment rates, experience in dealing with previous pandemics such as HIV, and the resilience and agility levels of those in the global South that navigate crisis on a daily basis, are rich areas for further scholarly work.

## Author contributions

All authors contributed equally to the manuscript revision, read, and approved the submitted version.
